# A comparative study of progressive failure of granite and marble rock bridges under direct shearing

**DOI:** 10.1038/s41598-024-61605-2

**Published:** 2024-05-13

**Authors:** Guangming Luo, Shengwen Qi, Bowen Zheng

**Affiliations:** 1 Key Laboratory of Shale Gas and Geoengineering, Institute of Geology and Geophysics, Chinese Academy of Sciences, Beijing, 100029 China; 2 College of Earth and Planetary Sciences, University of Chinese Academy of Sciences, Beijing, 100049 China; 3 Innovation Academy for Earth Science, Chinese Academy of Sciences, Beijing, 100029 China

**Keywords:** Direct shearing, Rock bridges, Progressive failure, Normal stress effect, Lithology effect, Solid Earth sciences, Engineering

## Abstract

Shear failure of rock bridges is an important process in geological phenomena, including landslides and earthquakes. However, the progressive failure of natural rock bridges has not yet been fully understood. In this work, we carried out direct shearing experiments on both granite and marble rock bridges, and applied acoustic emission (AE) monitoring throughout the experiments. With the mechanical curves and the evolution of AE activity (including AE energy rate and *b* value), the failure of rock bridges can be divided into three pre-failure phases and one ultimate failure phases. We analyzed the effects of normal stress and lithology on the pre-failure phases. We noted that with the increasing of normal stress, the length of stable cracking phase decreases and the length of unstable cracking phase slightly increases, except for marble rock bridges at high normal stress, which maintains a great proportion of stable cracking phase that possibly results from the great off-fault damage. Increasing normal stress also suppresses the dilation of granite rock bridges, but has a different effect on marble rock bridges, which also suggests the effect of lithology on failure modes.

## Introduction

Rock bridge is an intact or strong segment separating coplanar or non-coplanar discontinuities^1^. Shear failure of rock bridges is a critical process leading to failure of rock masses such as landslides^2^ and earthquakes^3–5^. Laboratory experiments provide the main approach for establishing the mechanical properties of rocks. Recently, shear apparatuses have been developed for shear tests on rock mass, e.g., the dynamic direct shear testing device developed by Qi et al. (2020)^6^ and the multifunctional shear apparatuses developed by Zhao et al. (2023, 2024)^7,8^. Experimental studies have revealed the effect of normal stress, joint geometry or persistence rate (the ratio of joint length to the overall length of discontinuity) on the direct shearing properties of rock bridges^9–15^. However, the experiments with natural rock samples are limited, e.g., Miao et al. (2024) recently studied the directing shearing behaviors of sandstone rock bridges under different normal stresses^11^. The effect of lithology on the direct shearing failure of rock bridges is still poorly understood.

It has been widely accepted that when subjected to loading, rock sample loses it strength in a progressive failure process, including phases of (1) crack closure, (2) linear elastic deformation, (3) stable cracking, (4) unstable cracking, (5) ultimate failure and post-peak behavior^16–18^. The phases have been identified mainly by the stress–strain characteristics displayed through axial and lateral deformation measured in laboratory compression tests^16,17,19–21^, and the corresponding stress threshold for dividing these phases, such as crack initiation stress *σ*_ci_ and crack damage stress *σ*_cd_ can be defined by crack volumetric strain such as in Martin and Chandler (1994)^20^. For direct shearing on rock bridges, volumetric strain is hard to be calculated due to the loading mode and thus stress thresholds cannot be defined by the direct measurement of deformation.

As an indirect measure of the damage accumulated in rock volume by cracking, acoustic emission (AE) technique has been applied to help characterize the progressive failure of rock^22–25^. The heterogeneity imposed by crystalline rocks such as granite has been found to have a significant effect on the strength and deformation responses and the associated microcracking behavior of rocks^26^. Therefore, AE technique provides an important tool to characterize the lithology effect on the failure process of rock. By far, the comparison of AE evolution between rock bridges with different lithology has not been reported.

In this study, we comparatively studied the direct shearing failure of granite and marble rock bridges, and applied the AE monitoring during the direct shearing experiments. The mechanical curves and the evolution of AE activity during shearing help characterize the progressive failure phases and the corresponding stress thresholds. The effects of normal stress and lithology on the pre-failure phases have been analyzed and the failure mode under different lithology has been discussed.

## Methodology

**Figure 1:**
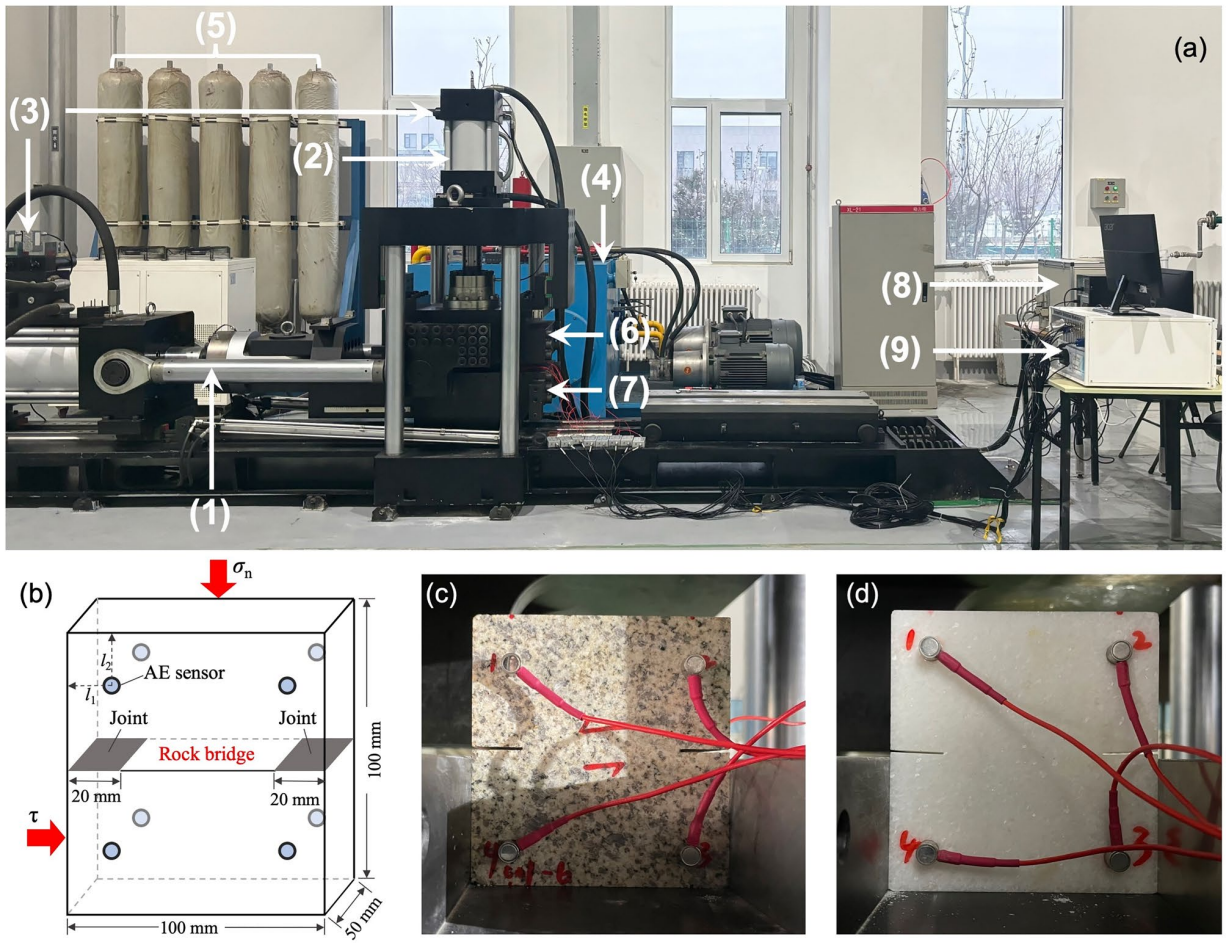
(**a**) Photograph of the dynamic direct shear testing device: (1) tangential hydrocylinder, (2) normal hydrocylinder, (3) servo valve, (4) oil source, (5) accumulator, (6) upper shear box, (7) lower shear box, (8) Computer and control device, and (9) PAC acoustic emission device. (**b**) Sketch of the rock bridge sample with the AE sensors (*l*_1_ = 15 mm, *l*_2_ = 20 mm). (**c**) Granite rock bridge sample. (**d**) Marble rock bridge sample.

We acquired granite and marble samples without fractures and bedding from quarries in Suizhou City, Hunan Province and Hezhou City, Hubei Province of China, respectively. Cylindrical specimens with dimensions of *Φ* 50 × 100 mm were prepared for uniaxial compression tests to obtain the basic mechanical properties. Brazilian splitting tests were also implemented to obtain the tensile strength of disc specimens with sizes of *Φ* 50 × 25 mm. The basic mechanical properties of the samples are listed in Table 1. Rock bridge specimens were made by cutting rock blocks into cubic rock specimens with dimensions of length 100 mm, width 50 mm and height 100 mm (Fig. 1b). Using high-pressure waterjet, two joints with an aperture of 1 mm were cut at the middle height of the specimen. The length of joints was set as 20 mm, leaving the central region as the rock bridge (60 mm).

**Table 1:** The basic mechanical properties of the granite and marble sample.

Lithology	Density (g/cm^3^)	Uniaxial compression strength (MPa)	Tensile strength (MPa)	Young’s modulus (GPa)	Poisson’s ratio
Granite	2.59	128.81	6.69	30.86	0.27
Marble	2.46	123.73	6.67	26.16	0.21

Direct shearing experiments are carried out using a dynamic direct shear testing device (Fig. 1a) at the Institute of Geology and Geophysics, Chinese Academy of Sciences (IGGCAS). Detailed description about this shearing device can be found in Qi et al. (2020)^6^. Constant normal stresses of 2 MPa, 4 MPa, 6 MPa are applied during each shear experiment, respectively. The normal stress is calculated by dividing the normal force by the whole area of the shear plane. Thus, the effective normal stress on the rock bridge is approximately 3.33–10 MPa, calculated by dividing the normal force by the area of rock bridge. The shear rate was set as 0.001 mm/s in all experiments, and the real-time values of normal load, shear load, normal displacement, and shear displacement were recorded during the experiment. We terminated the experiments at the shear displacement of 2 mm, ensuring that all the samples could be loaded to failure.

During the direct shearing, AE monitoring was carried out using an AE system manufactured by PAC (Physical Acoustic Corporation) (Fig. 1a). Eight Nano-30 sensors are attached onto the sample by superglue (Fig. 1c, d). AE data is acquired during the direct shear experiments with hit-based streaming method^27^ with a sampling rate of 2 MHz and a threshold of 35 dB to exclude the noise. We summed up the energy of each AE hit per second to calculate the AE energy rate, which acts as a proxy for the number and relative size of microcracks^28^. We also calculated the *b* value to analyze the size distribution of AE events as Eq. (1).

1 $${\text{log}}{\text{N}}\,\left({>}{\text{A}}\right)\,{=}\,{\text{C}}-{\text{b}} \, {(}{\text{A}}{/20)}{,}$$

where *A* is the amplitude of AE events (unit: dB), *N*(> *A*) is the number of events that has an amplitude larger than *A*. *C* and *b* is the coefficients. To obtain the variations in *b* value during the progressive failure of rock bridges, we applied a sliding window covering 1500 events and a sliding step of 500 events. In each sliding window, the *b* value was obtained from the cumulative distribution of the AE amplitude as in Eq. (1), fitted in a Least Squares method. The slope of this fitting curve gave the b value.

## Results

### Mechanical curves and failure patterns

**Figure 2:**
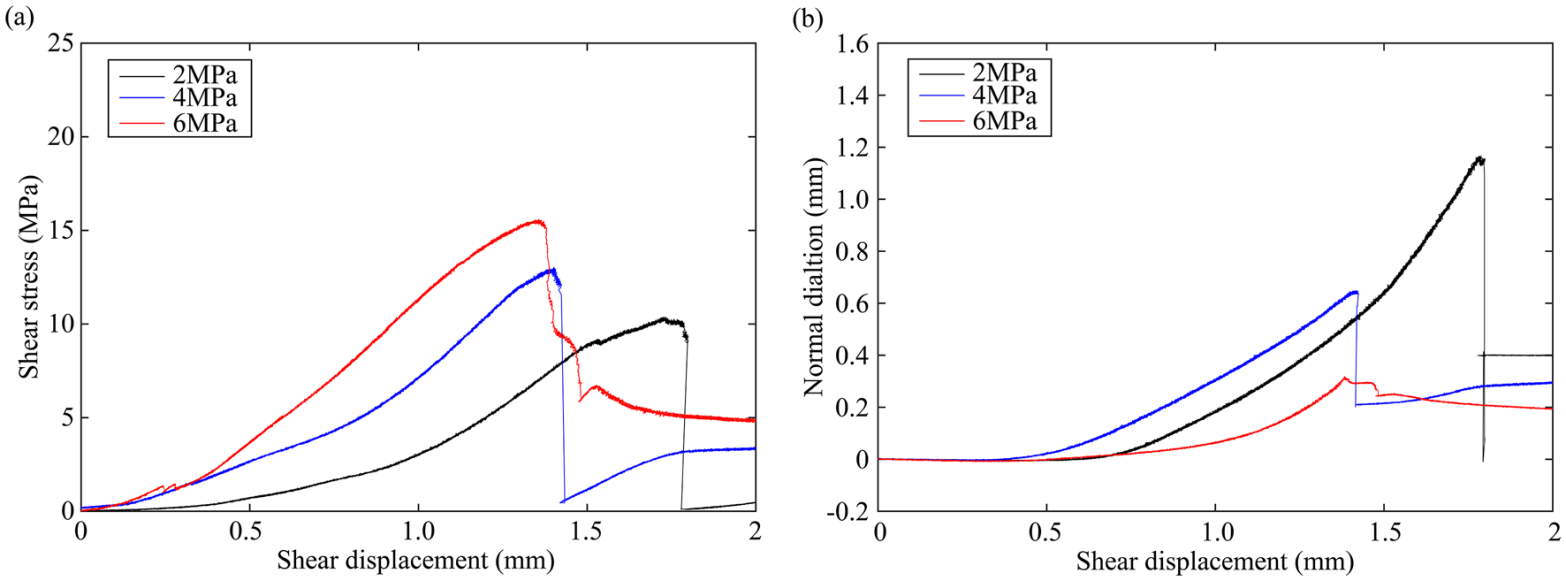
Mechanical curves of granite rock bridges. (**a**) Shear stress–shear displacement curves. (**b**) Normal dilation-shear displacement curves.

**Figure 3:**
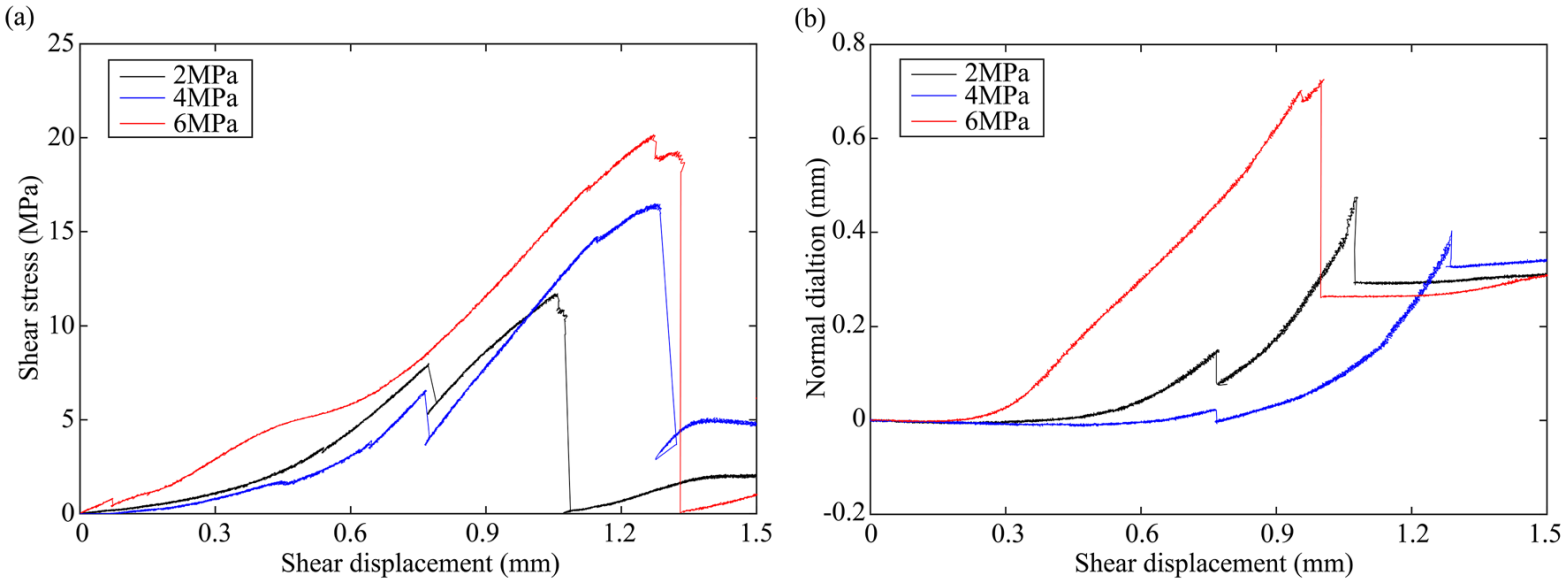
Mechanical curves of marble rock bridges. (**a**) Shear stress–shear displacement curves. (**b**) Normal dilation-shear displacement curves.

Figures 2 and 3 show the shear stress and normal dilation as a function of shear displacement for granite rock bridges and marble rock bridges, respectively. Shear stress-displacement curves of rock bridges show similar characteristics to that obtained in compression tests^29^: an initial increase in slope of the curve, followed by bending-over of the curve with stress-hardening which diminishes in slope is observed before the peak stress and the ultimate stress drop. The initial toe of the curve is generally attributed to the closure of pre-existing cracks^16^. The normal displacement first decreases to a lowest value and then continuously increases until it suddenly drops with the breakdown of rock bridge. The dilation rate is increasing before failure. It can be seen that increasing normal stress improves the peak strength for both granite and marble, and decreases the dilation for granite. However, the dilation at normal stress of 6 MPa is the largest among marble samples.

**Figure 4:**
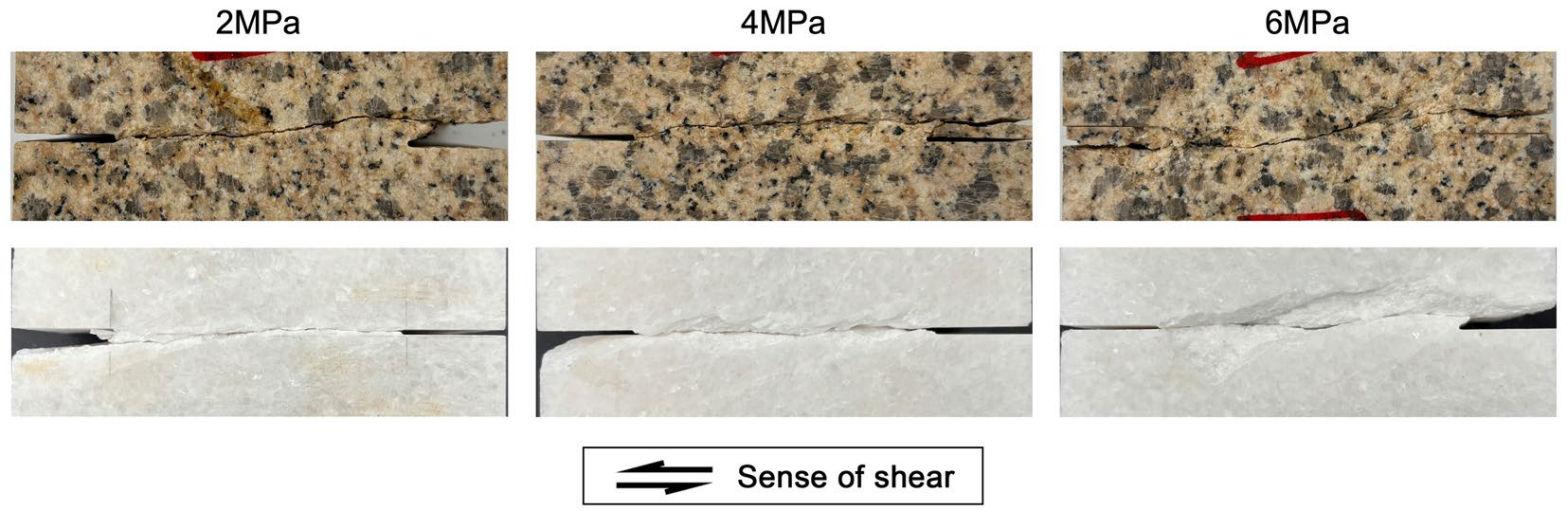
The coalescence patterns of rock bridges.

**Figure 5:**
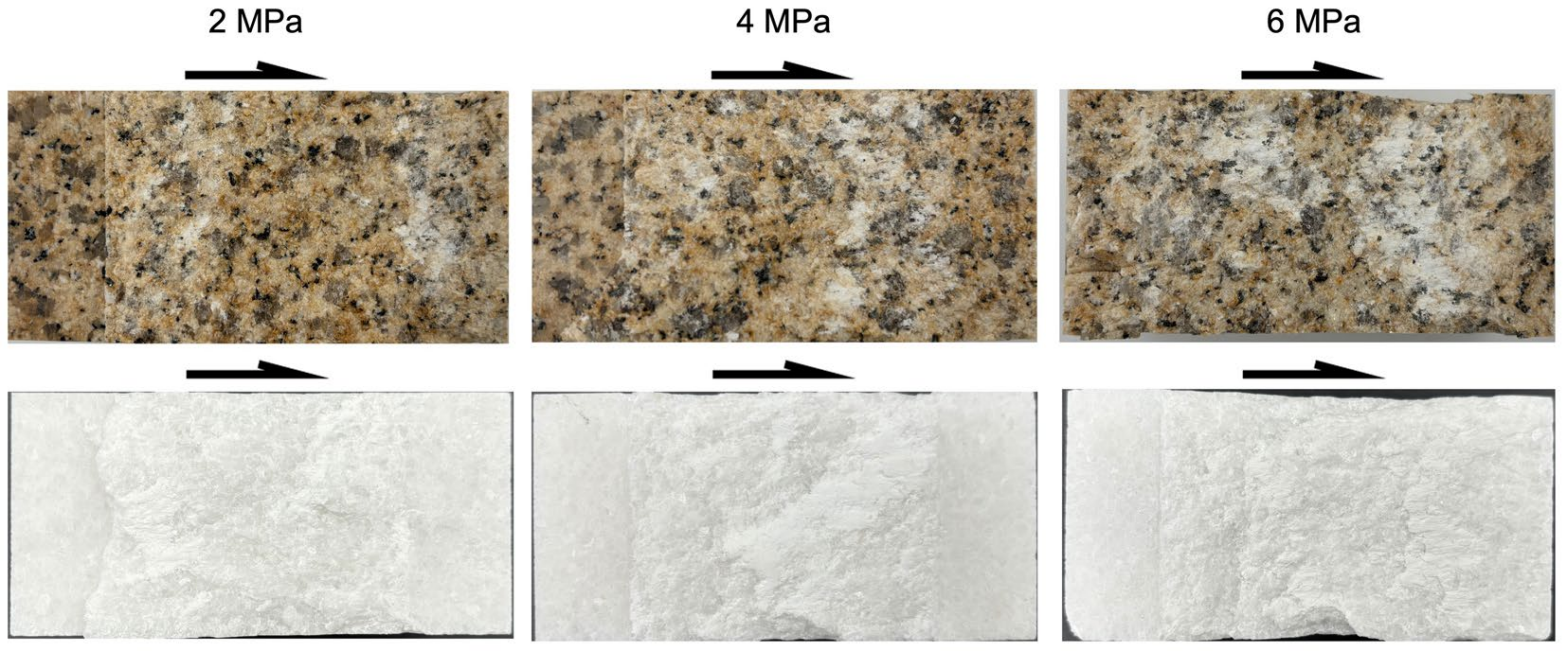
The failure surfaces of rock bridges (the arrows indicate the direction of movement of the opposite blocks).

As shown in Figs. 4 and 5, the coalescence patterns of granite rock bridges are all curved. Increasing normal stress seems not to alter the failure mode, but promote the compressive shearing, which is indicated by the increasing striations on failure surfaces. For marble rock bridges, the coalescence patterns are all flat. With the increasing of normal stress, more surface spalling and off-damage (indicated by the increasing width of white patches) can be observed especially at high normal stress of 6 MPa. The failure surfaces of marble rock bridges at all normal stresses show clear striations. At normal stress of 6 MPa, the failure surface is significantly undulated.

### Progressive failure phases

**Figure 6:**
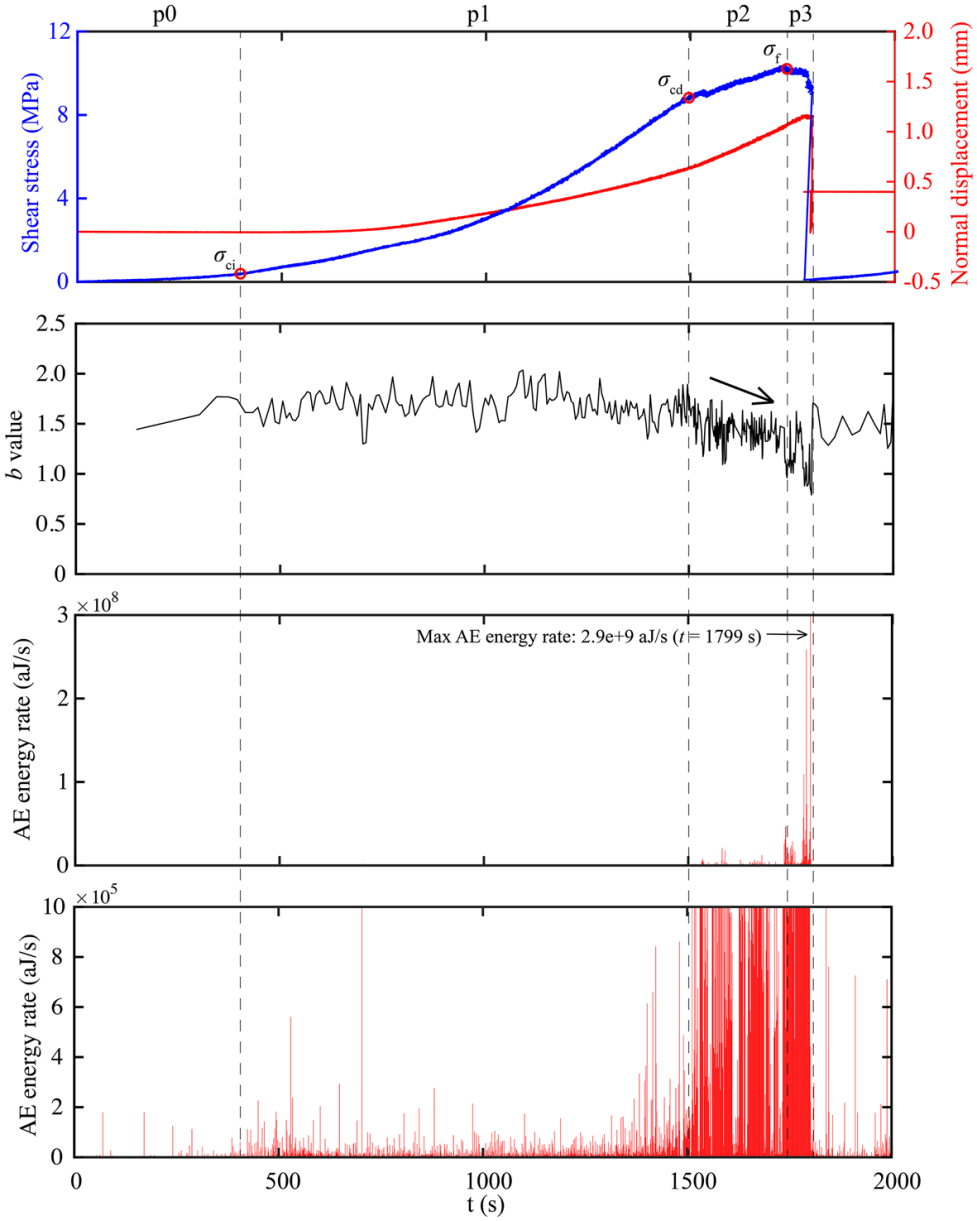
The mechanical curves and the corresponding evolution of AE activities (taking the granite rock bridge at the normal stress of 2 MPa as an example).

To identify the progressive failure phases of rock bridges under direct shearing, we analyze both the mechanical curves and the evolution of AE activities during shearing. As in Fig. 6, at the beginning of loading, the sample was first compressed slightly due to the crack closure (phase p0). After that, it begins to dilate. Different with compression test^16^, there is not a notable ‘linear elastic deformation’ phase between *σ*_cc_ (crack closure stress) and *σ*_ci_ where the dilation curve remains flat. We therefore define the *σ*_ci_ at the onset of dilation, corresponding to the end of crack closure and the beginning of cracking. Before reaching the peak stress, the failure progress can be further divided into two pre-failure phases according to the AE activity. The size-frequency distribution of AE events keeps unchanged as *b* value fluctuates around the same level, and with a smaller number of events at the first phase p1, while the *b* value shows obvious decrease and the number of events increases significantly at the second phase p2 (indicated by the dense data points of *b* value). In addition to the decrease of *b* value, there is also a rapid increase of AE energy rate at the transition between p1 and p2. In a broad sense, similar acceleration behaviors have been observed preceding catastrophic rupture of rock on both the laboratory scale and the fault scale (also described as the “finite-time singularity”) ^30^. These two signs help us define the stress threshold *σ*_cd_, which suggests that beyond this stress threshold, the rock sample will undergo unstable cracking due to the damage accumulated in rock volume^20^. Therefore, we define phases p1 and p2 as the stable cracking phase and unstable cracking phase, respectively. The unstable cracking phase is ended at the peak stress *σ*_*f*_. After that, there is an ultimate failure phase p3 characterized by the post-peak stress-weakening and stress drop.

Using the above method, the stress thresholds and the corresponding shear displacement for granite and marble rock bridges under different normal stress can be obtained as listed in Table 2. We can see that for direct shearing of rock bridges, the *σ*_ci_ is smaller than 0.1*σ*_*f*_, which is less than 0.3–0.4*σ*_*f*_ that generally observed in compression experiments. The relative smaller *σ*_ci_ of rock bridges might result from the smaller proportion of rock volume involved in the crack closure phase compared to the compression experiments. The *σ*_cd_ is about 0.8*σ*_*f*_ for samples of our experiments, which is similar to compression experiments^31^.

**Table 2:** The stress thresholds and the corresponding displacement.

Lithology	*σ*_n_ (MPa)	*d*_ci_ (mm)	*σ*_ci_ (MPa)	*d*_cd_ (mm)	*σ*_cd_ (MPa)	*d*_p_ (mm)	*σ*_p_ (MPa)	*σ*_ci_* /σ*_p_	*σ*_cn_* /σ*_p_
Granite	2	0.40	0.39	1.50	8.96	1.73	10.38	0.04	0.86
	4	0.25	1.01	1.22	10.69	1.40	13.04	0.08	0.73
	6	0.29	1.25	1.05	12.17	1.36	15.58	0.08	0.78
Marble	2	0.14	0.39	0.92	9.02	1.05	11.69	0.03	0.77
	4	0.38	1.27	1.08	13.05	1.28	16.48	0.06	0.79
	6	0.09	1.38	1.11	17.75	1.27	20.86	0.07	0.85

**Figure 7:**
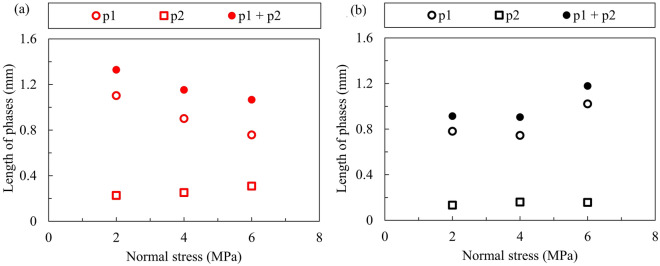
The length of pre-failure phases p1 and p2 for (**a**) granite and (**b**) marble rock bridge.

**Figure 8:**
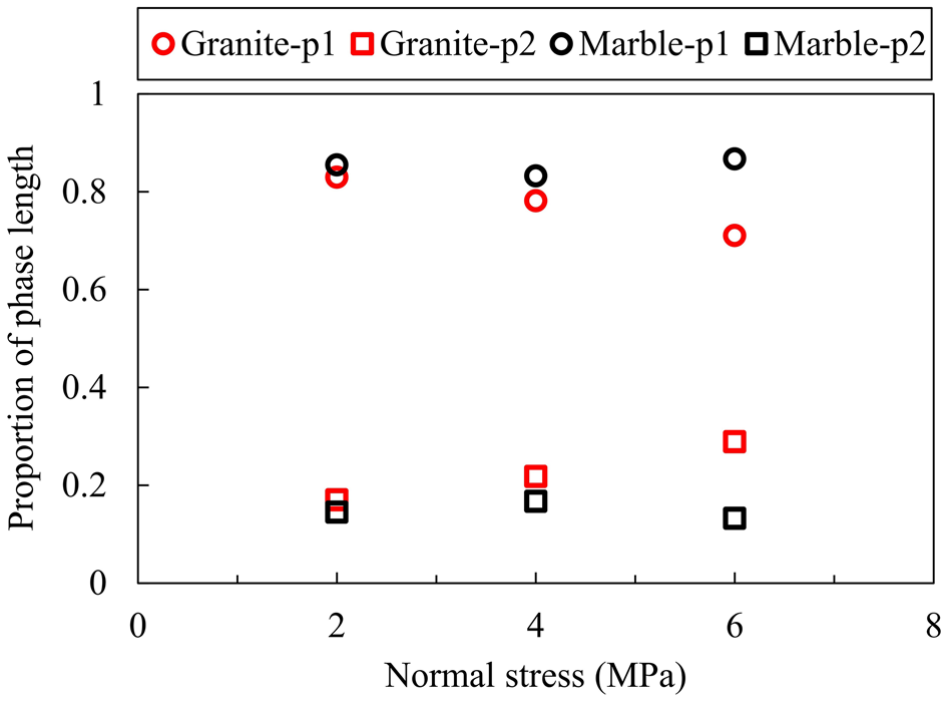
The proportion of pre-failure phases (normalized by the total length of p1 and p2).

For granite rock bridges, we can see from Fig. 7 that the total length of the pre-failure phases (p1 + p2) and the length of p1 decrease with increasing normal stress, while the length of p2 shows a slightly increasing trend. However, for marble granite, a similar trend can only be seen when the normal stress increases from 2 to 4 MPa, and an intriguing increase of length of p1 is observed at the normal stress of 6 MPa. Thus, the total length of the pre-failure phases is greatest at normal stress of 6 MPa for marble rock bridges. In addition to the absolute length of pre-failure phases, the proportion of these phases also shows a different effect of high normal stress between granite and marble rock bridges. As shown in Fig. 8, it is easily noted that the proportion of p1 decreases and the proportion of p2 increases with increasing normal stress for granite rock bridges, while the marble rock bridge maintains a high proportion of p1 at normal stress of 6 MPa. The length of the stabling cracking phase p1 is about 4 times the length of the unstable cracking phase p2 except for marble at the normal stress of 6 MPa.

### Dilation

**Figure 9:**
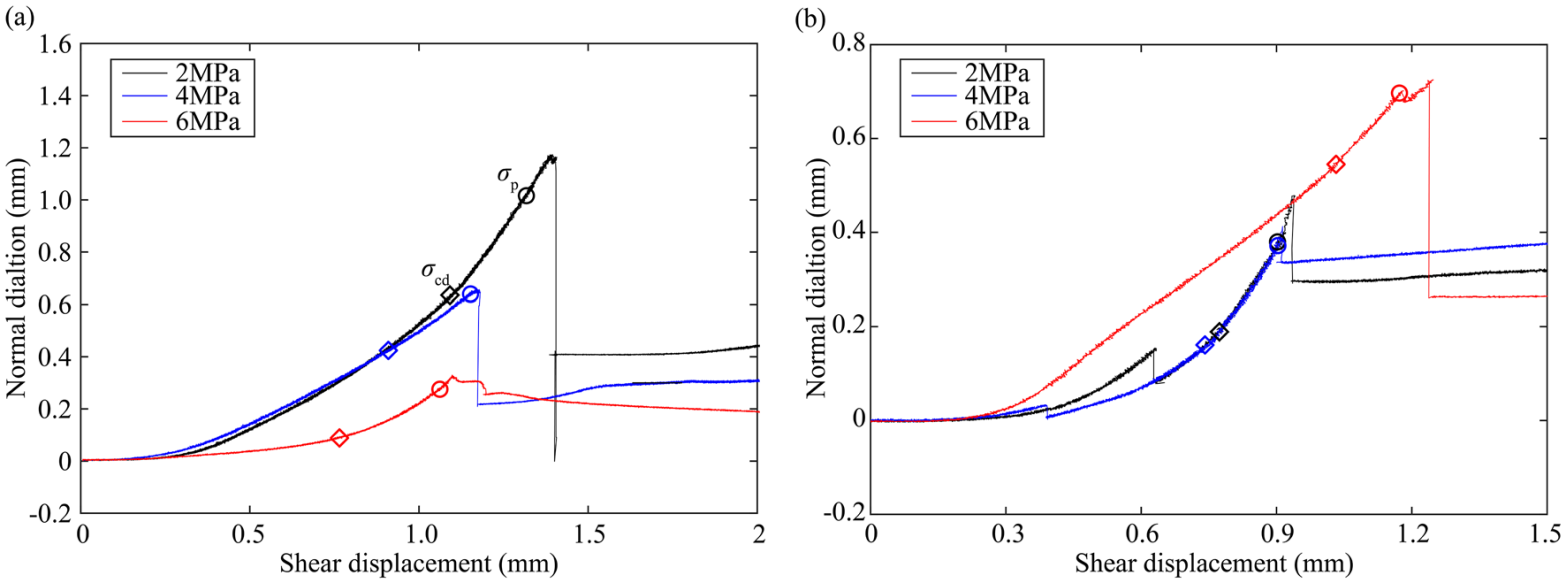
The normal dilation curves of (**a**) granite and (**b**) marble rock bridges (shear displacement is re-calculated from the onset of dilation).

**Figure 10:**
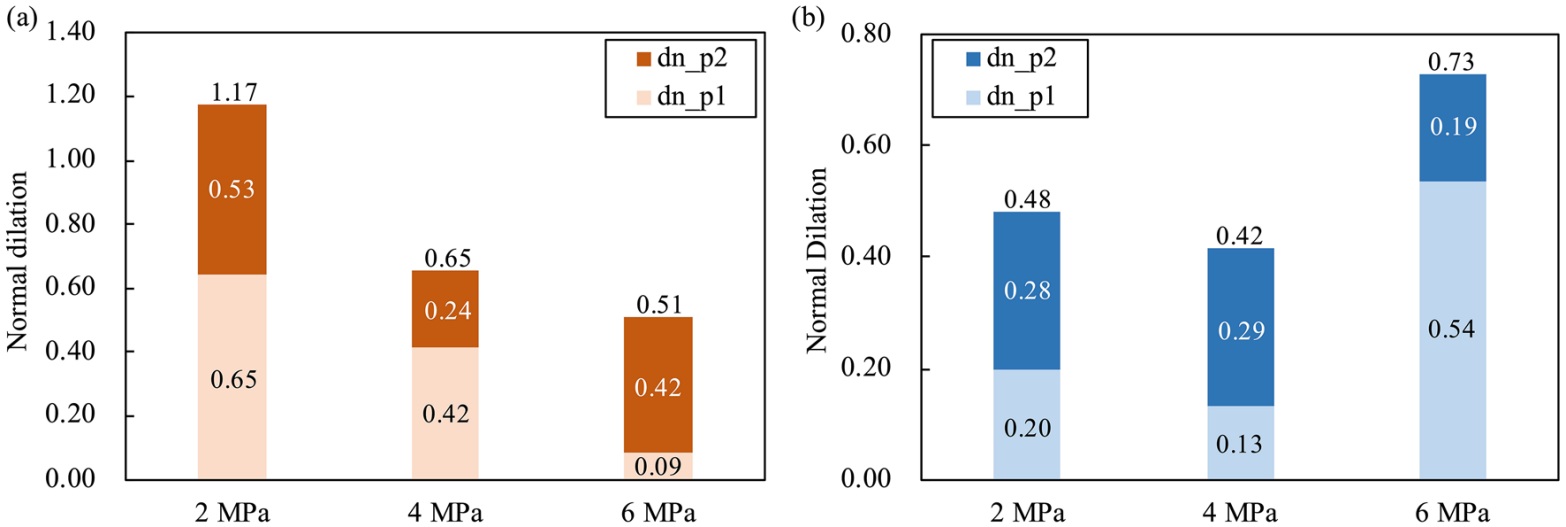
The normal dilation of (**a**) granite and (**b**) marble rock bridges in pre-failure phases p1 and p2 (dn_p1 and dn_p2 denote the normal dilation during the phases p1 and p2, respectively).

Dilation of rock under stress is the result of cracking in rock volume (Brace et al., 1966). As shown in Figs. 9 and 10, we compare the normal dilation of granite and marble rock bridges by re-calculating the shear displacement from the onset of displacement (i.e., the *d*_ci_). For granite rock bridges, the dilation during p1 (dn_p1) and the total pre-failure dilation (dn_p1 + dn_p2) both decrease when the normal stress increases (consistent with the compressive shearing properties of the failure surfaces in Fig. 5). For marble rock bridges, the dilation during p1 and the total pre-failure dilation show limited variations when the normal stress increases from 2 to 4 MPa, but at the normal stress of 6 MPa, the dilation during p1 shows a significant increase and thus increases the total pre-failure dilation.

## Discussions on the effects of normal stress and lithology

From Figs. 7 and 10, we can tell that the normal stress exerts its effect on the failure progress of rock bridges mainly by the stable cracking phase p1. For granite rock bridges, the decrease of length of p1 and the decrease of dilation in p1 are obvious when the normal stress increases. The curved coalescence patterns and the failure surfaces of granite rock bridges at all normal stresses (Figs. 4 and 5) indicate the same mode of macroscopic tensile failure, consistent with previous experimental works on granite rock bridges with similar conditions of persistence, normal stress and shear rate^14,32^. According to Lajtai’s tensile failure criteria on rock bridge, the inclination of macroscopic tensile fracture would get smaller when the normal stress increases^9^. Thus, on one hand, this resulted in less dilation under high normal stress. On the other hand, the damage is more concentrated on the shear plane due to the stress concentration. With limited volume of rock bridge subject to stress, the sample could be easier to be loaded to unstable cracking and therefore the proportion of p1 is getting smaller.

For marble rock bridges, the flat coalescence patterns and striations indicate the same mode of macroscopic shear failure along the shear plane (Figs. 4 and 5). This agrees with the relatively less dilation of marble samples than granite sample (Fig. 10). At normal stress of 6 MPa, a high proportion of p1 and dilation in p1 are observed, compared to those at low normal stress. This could be explained by the great off-fault damage indicated from its undulated failure surface (Fig. 5). The more volume of rock being damaged, the longer the stable cracking phase will last, as more deformation could be modulated through cracking until it develops into the unstable cracking phase.

The different failure modes of granite and marble rock bridges in our experiments, namely the macroscopic tensile failure (curved coalescence pattern) and shear failure (straight coalescence pattern) respectively, are also observed in the experiments of Tham et al. (2005) under uniaxial tension^33^, which have been attributed to the difference in heterogeneity of rock fabric^34^. As crystalline rock, granite contains different types of minerals. Based on the micro-photograph of thin sections observed under a polarizing microscope, the granite sample in our experiments consists of feldspar, quartz and hornblende, and the grain size ranges from 0.2 to 3 mm, while carbonate (the grain size is about 2 mm) is almost the only component for our marble sample (metamorphic rock). According to the modeling of Wang et al. (2016)^35^, more homogeneous fabric would significantly promote the shear localization under loading, and thus leads to a failure mode of macroscopic shear failure. For sandstone rock bridges, Miao et al. (2024) carried out direct shear experiments and they have also observed a similar normal stress effect on the coalescence of rock bridges and the length of p2 with our results of granite samples (the variation of p1 is unknown in their experiments as *σ*_ci_ has not been identified)^8^. To obtain a comprehensive understanding of the progressive failure of rock bridges, more experiments under different conditions of lithology, normal stress should be further carried out.

In natural fault zones, similar effects of normal stress on the deformation localization and the dilation processes have also been observed^11,36^. Therefore, the progressive failure phases of rock bridges and the corresponding normal stress effect revealed by our experiments could be in principle up-scaled to faulting processes, and the scale effect of rock bridges should be taken into account.

## Conclusions

We conducted direct shearing experiments on granite and marble rock bridges, and defined the progressive failure phases and the corresponding stress thresholds based on the mechanical curves and the evolution of AE activity during shearing. The effects of normal stress and lithology on the failure phases were analyzed. The main conclusions are as follows:

1. The failure process of granite and marble rock bridges could be divided into four phases: crack closure phase p0, stable cracking phase p1, unstable cracking phase p2 and ultimate failure phase p3. The stress threshold *σ*_ci_ is less than 10% *σ*_f_, and the *σ*_cd_ is about 80% *σ*_f_. The length of the stabling cracking phase p1 is about 4 times the length of the unstable cracking phase p2 except for marble at the normal stress of 6 MPa.
2. With the increasing of normal stress, the length of p1 decreases and the length of p2 slightly increases for granite rock bridges. However, this variation trend can only be observed when normal stress increases from 2 to 4 MPa for marble rock bridges. For marble at the normal stress of 6 MPa, a significant length of p1 was observed, which may be explained by the great off-fault damage.
3. For granite rock bridges, the dilation during phase p1 and the total pre-failure dilation both decrease when the normal stress increases. For marble rock bridges, the dilation shows limited variations when the normal stress increases from 2 to 4 MPa, but at the normal stress of 6 MPa, the dilation during p1 shows a significant increase. The normal stress effect on the dilation is consistent with the failure modes of rock bridges.

## Data Availability

Data supporting the results of the study can be accessed upon reasonable request from the corresponding author.
